# Ocular surface complications following biological therapy for cancer

**DOI:** 10.3389/ftox.2023.1137637

**Published:** 2023-06-22

**Authors:** Kevin Sheng-Kai Ma, Ping-Feng Tsai, Tina Yi-Jin Hsieh, James Chodosh

**Affiliations:** ^1^ Department of Epidemiology, Harvard T.H. Chan School of Public Health, Boston, MA, United States; ^2^ Center for Global Health, Perelman School of Medicine, University of Pennsylvania, Philadelphia, PA, United States; ^3^ Division of Pharmacoepidemiology and Pharmacoeconomics, Department of Medicine, Brigham and Women’s Hospital, Harvard Medical School, Boston, MA, United States; ^4^ Department of Ophthalmology, Tri-Service General Hospital, Taipei, Taiwan; ^5^ Department of Environmental Health, Harvard T.H. Chan School of Public Health, Boston, MA, United States; ^6^ Department of Obstetrics and Gynecology, Beth Israel Deaconess Medical Center, Boston, MA, United States; ^7^ Department of Ophthalmology and Visual Sciences, University of New Mexico School of Medicine, Albuquerque, NM, United States

**Keywords:** biological therapy, cancer, cornea, immunotherapy, ocular surface, targeted therapy

## Abstract

Novel and highly effective biological agents developed to treat cancer over the past two decades have also been linked to multiple adverse outcomes, including unanticipated consequences for the cornea. This review provides an overview of adverse corneal complications of biological agents currently in use for the treatment of cancer. Epidermal growth factor receptor inhibitors and immune checkpoint inhibitors are the two classes of biological agents most frequently associated with corneal adverse events. Dry eye, Stevens-Johnson syndrome, and corneal transplant rejection have all been reported following the use of immune checkpoint inhibitors. The management of these adverse events requires close collaboration between ophthalmologists, dermatologists, and oncologists. This review focuses in depth on the epidemiology, pathophysiology, and management of ocular surface complications of biological therapies against cancer.

## Introduction

The emergence of biologicals as antineoplastic therapies began in the 1990s. Such agents inhibit the growth and survival of cancer cells, but can also induce severe side effects that affect multiple body systems. The various cell types present in the cornea each have distinct receptor expression profiles that makes the cornea susceptible to adverse outcomes during use of biological agents. This review will summarize the corneal complications of biological agents used in oncology and discuss the pathogenesis and clinical management of these adverse events.

## Tyrosine kinase inhibitors

Tyrosine kinases regulate cell proliferation and apoptosis by transducing intracellular signaling cascades. Their inhibitors, known as tyrosine kinase inhibitors (TKi), include agents that can suppress uncontrolled cell proliferation in various types of cancer. As the use of TKi to treat cancer has increased in recent years, awareness of ocular side effects from TKi has also increased. Among all TKi in clinical oncology practice, epidermal growth factor receptor inhibitors (EGFRi) have been most commonly reported to be associated with keratitis (Saint-Jean et al., 2018).

Epidermal growth factor receptors (EGFRs) are highly expressed on the ocular surface and periocular tissues, and adverse effects of EGFR inhibition on the cornea should not be surprising. Breakdown of the corneal epithelial barrier is often an initial harbinger of keratitis. Reduced epithelial cell proliferation in the cornea during EGFRi treatment results in loss of epithelial regeneration, impaired healing from environmental exposures such as dryness and exposure to particulate matter, and ultimately leads to corneal inflammation. Inhibition of the EGFR cascade also disrupts hair follicle growth cycle, resulting in trichomegaly which can add insult to the cornea due to trichiasis. Suppression of EGFR also inhibits the proliferation and repair of the meibomian glands. When meibum secretion is diminished, the tear film evaporates more rapidly, further compromising corneal epithelial repair (Ho et al., 2013; Huillard et al., 2014).

One such EGFRi, cetuximab, has been strongly associated with induction of keratitis (Table 1). Cetuximab is now approved to treat metastatic colorectal cancer and head and neck squamous cell carcinoma. Trichomegaly, conjunctivitis, and blepharitis are the most common ocular side effects reported as associated with cetuximab (Fraunfelder and Fraunfelder, 2012). According to post-marketing surveillance in Japan, the incidence of ocular adverse events linked with cetuximab was approximately 2.6%, and the severity of most adverse events was less than grade 2 (Ishiguro et al., 2012; National Institutes of Health, 2022). However, in select case reports, cetuximab was associated with severe keratitis (Specenier et al., 2007).

**TABLE 1:** Biological anti-cancer agents–their indications and reported ocular adverse effects.

Drug	Indications	Corneal adverse event
Cetuximab	Colorectal cancer, squamous cell carcinoma of the head and neck	Conjunctivitis
Afatinib	Non-small cell lung cancer	Ulcerative keratitis
Erlotinib	Non-small cell lung cancer, pancreatic cancer	Trichomegaly, keratitis
Gefitinib	Non-small cell lung cancer	Keratitis
Nivolumab	Melanoma	Dry eye, keratitis
Pembrolizumab	Non-small cell lung cancer	Dry eye

Afatinib is another EGFRi that is now used as a first-line treatment for non-small cell lung cancer. Common side effects associated with afatinib include dry eye and ulcerative keratitis (McKelvie et al., 2019). In the LUX-lung 3 trial for metastatic lung adenocarcinoma with *EGFR* mutations (Sequist et al., 2013), the prevalence of keratitis in these patients was 2.2%. Notably, approximately 0.4% of patients had grade 3 keratitis, leading to the discontinuation of the therapy (Yang et al., 2015). Cases of trichomegaly and keratitis have also been reported with other EGFRi, for example, erlotinib and gefitinib, as well as other TKi (Zhou et al., 2016; Rawluk and Waller, 2018).

Artificial tears and lubricating ointments are frequently used to protect and rehydrate an injured corneal epithelium. Additionally, topical corticosteroids can be applied to block inflammation, which may confer rapid relief of pain (Huillard et al., 2014). In large epithelial defects, bandage contact lenses may be prescribed to protect the cornea and alleviate pain. However, if TKi-associated keratitis proves unresponsive to these measures, it may be necessary to discontinue the EGFRi (Johnson et al., 2009). Treatment of such cases with EGF-containing eyedrops is a unique approach still not validated in a human clinical trial. However, Kawakami et al. reported dramatic improvement associated with starting topical human recombinant EGF in a patient with severe filamentous keratitis after beginning cetuximab treatment for colorectal cancer. The keratitis cleared just 3 weeks after starting topical recombinant EGF, despite continuation of the cetuximab (Kawakami et al., 2011). EGFR inhibitors have also been found to induce skin toxicity through upregulating keratinocyte cytokine release (CCL2, CCL5, CCL27, and CXCL14) that leads to chemokine-driven skin inflammation, which may deter patients from taking the medication (Lichtenberger et al., 2013). Nonetheless, skin toxicity can also be an important predictor of drug response, making it difficult for clinicians to decide whether to discontinue treatment due to cutaneous and/or ophthalmological side effects, which requires collaboration between medical specialties.

## Immune checkpoint inhibitors

Immune checkpoints occur when costimulatory T cell receptors bind to “checkpoint” proteins on the surface of tumors that results in sending an “off” signal to the T cells, thus reducing host immune responses to the cancer. Immune checkpoint inhibitors (ICI) are agents that block this process, thus rendering tumors susceptible to host immune attack. The development of ICI has greatly benefitted the progression-free survival and in select instances, the rate of cure for patients with what were previously difficult or frankly untreatable malignancies, often including metastatic disease (Goleva et al., 2021). The primary checkpoint proteins targeted in this pathway are cytotoxic T-lymphocyte-associated protein 4 (CTLA-4), and programmed cell death protein 1(PD-1), along with the PD-1 binding partner, programmed death ligand 1 (PD-L1). However, cutaneous, neurological, cardiac, and ocular adverse events, the latter including ocular myasthenia, uveitis, and dry eye, have been associated with ICI therapy since their introduction as treatment for multiple types of cancer (Vanhonsebrouck et al., 2020; Huang et al., 2021; Park et al., 2021; Chiang et al., 2022a; Chiang et al., 2022b; Kao et al., 2022; Lee et al., 2022).

Dry eye affects between 1% and 24% of patients on ICI. The mechanism for dry eye in persons on ICI therapy, as proposed by Hiro et al., is thought to be loss of self-tolerance and induction of autoimmunity, resulting in primary lacrimal dysfunction and clinical sicca syndrome (Hori et al., 2020). A similar mechanism has been proposed for the cornea with disruption of immune privilege, and subsequent T cell infiltration at the ocular surface. Among U.S. Food and Drug Administration (FDA)-approved ICI, nivolumab and pembrolizumab had the highest incidence of ocular adverse effects, followed by atezolizumab and ipilimumab (Fang et al., 2019; Hori et al., 2020) (Table 1).

During the phase II trial of nivolumab in subjects with ipilimumab-refractory melanoma, three patients (3%) treated with 3 mg/kg nivolumab suffered either grade 1 or 2 dry eye (Weber et al., 2016). In the KEYNOTE-010 clinical trial, which compared pembrolizumab to docetaxel for previously treated, PD-L1-positive, advanced non-small-cell lung cancer, out of 1,034 study subjects, ten (1.5%) treated with pembrolizumab experienced grade 1, 2 dry eye, while only one person treated with docetaxel reported dry eye (Herbst et al., 2016). While most documented dry eye occurrences are grade 1 or 2, there have been reports of more severe dry eye necessitating withdrawal of the ICI. A 58-year-old man with metastatic melanoma developed bilateral superficial punctate keratitis after receiving six courses of nivolumab treatment. Despite punctal plugs to increase the tear film, and use of topical cyclosporin, one cornea perforated. Three weeks following the withdrawal of nivolumab, and concurrent with institution of topical loteprednol (0.5%), and topical autologous serum, the perforation healed (Nguyen et al., 2016).

Treatment with ICI has also been associated with Stevens-Johnson syndrome/toxic epidermal necrolysis (SJS/TEN), which can result in severe cicatrizing keratoconjunctivitis leading to blindness. ICI-related SJS/TEN was reported in a case series involving eight individuals with SJS and an ALDEN score greater than four. Five patients exhibited ocular involvement, and three individuals exhibited grade 3 ocular involvement. Of the three patients with severe ocular involvement, two were being treated with pembrolizumab, and one with atezolizumab. In this series, patients with nivolumab-associated SJS/TEN exhibited little to mild ocular involvement (Ma et al., 2021).

ICI has also been hypothesized to be associated with corneal transplant rejection. An 85-year-old asymptomatic woman with a history of bilateral penetrating keratoplasty presented with bilateral diffuse keratic precipitates and subepithelial infiltrates 3 months after starting immunotherapy with pembrolizumab for a metastatic urothelial cell carcinoma. The corneal transplant rejection was treated with topical dexamethasone drops, but relapsed 2 weeks after the drops were discontinued. After consulting with an oncologist, pembrolizumab was discontinued (Vanhonsebrouck et al., 2020).

## Conclusion

As the clinical use of biological anti-cancer agents expands, the frequency of associated side effects is also expected to increase. This article briefly overviews corneal adverse effects associated with biological agents, particularly EGFRi and ICI. More research is needed to pinpoint the molecular basis for these adverse events. Ophthalmologists and other medical professionals should be aware of corneal adverse events in patients receiving biological agents for cancer. In order to prevent sight-threatening complications, the management of corneal adverse events requires close coordination between oncologists, ophthalmologists, and dermatologists so that the important benefits of anti-cancer therapies are balanced against the potential loss of vision in the small but significant number of treated patients who develop keratitis.
